# Enterovirus D68 Subclade B3 in Children with Acute Flaccid Paralysis in West Africa, 2016

**DOI:** 10.3201/eid2609.200312

**Published:** 2020-09

**Authors:** Amary Fall, Ndack Ndiaye, Kevin Messacar, Ousmane Kebe, Mamadou Malado Jallow, Hamid Harouna, Davy Evrard Kiori, Sara Sy, Déborah Goudiaby, Mohamed Dia, Mbayame Ndiaye Niang, Kader Ndiaye, Ndongo Dia

**Affiliations:** Institute Pasteur Dakar, Senegal (A. Fall, N. Ndiaye, O. Kebe, M.M. Jallow, D.E. Kiori, S. Sy, D. Goudiaby, M. Dia, M.N. Niang, K. Ndiaye, N. Dia);; University of Colorado, Aurora, Colorado, USA (K. Messacar);; Ministère de la Santé Publique, Niamey, Niger (H. Harouna)

**Keywords:** acute flaccid myelitis, acute flaccid paralysis, AFM, AFP, enterovirus D68, enteroviruses, EV-D68, polio surveillance, viruses, West Africa

## Abstract

We tested for enterovirus D68 in fecal samples collected during June–September 2016 from 567 patients with acute flaccid paralysis in 7 West Africa nations. Children <5 years old comprised 64.3% of enterovirus D68 positive patients. Our findings emphasize the need for active surveillance for acute flaccid myelitis.

Until 2014, enterovirus D68 (EV-D68) infections had been identified only sporadically after its discovery in 1962, but since 2014, the virus has emerged to cause large outbreaks of respiratory disease worldwide. In recent years, EV-D68 has been reported in outbreaks in the United States, Canada, Europe, Asia, and Africa, affecting >2,287 persons worldwide (*1*–*4*). 

The 2014 EV-D68 outbreak coincided temporally and geographically with increases in cases of acute flaccid myelitis (AFM), a subtype of acute flaccid paralysis (AFP), described by the Centers for Disease Control and Prevention as acute-onset flaccid weakness, combined with spinal cord lesions confirmed by magnetic resonance imaging, largely restricted to the gray matter, and spanning >1 spinal segments (*5*). In 2014, a total of 120 AFM cases in the United States (*4**,**6*) and >6 in Europe were associated with EV-D68 outbreaks. Subsequent biennial circulation of EV-D68 was associated with surges in AFM cases in the United States in 2016 and 2018 (*7*). In addition, 29 EV-D68–associated AFM cases were reported in Europe in 2016 (*8*). Africa, unlike Europe and the United States, has no active AFM surveillance. However, a 2016 study in Senegal reported 4 cases of paralysis associated with EV-D68 identified in fecal samples from children with AFP (*2*). With no AFM- or AFP-specific surveillance data available, we analyzed fecal samples collected for polio surveillance to better understand the extent of EV-D68 associated with AFP in West Africa and the genetic diversity of identified strains. 

## The Study 

We retrospectively tested for EV-D68 in fecal samples from patients <15 years old with AFP. The samples were collected during June–September 2016 as part of routine poliomyelitis surveillance in Niger, Senegal, Guinea, Mauritania, Gambia, Guinea-Bissau, and Cape Verde. Specimens were collected 24–48 hours apart and <14 days of paralysis onset. We inoculated fecal specimens onto human rhabdomyosarcoma cells after using chloroform for EV isolation according to the procedures described in the laboratory manual for the World Health Organization’s Global Polio Laboratory Network (http://polioeradication.org/wp-content/uploads/2017/05/Polio_Lab_Manual04.pdf). We used a QIAmp Viral RNA Mini Kit (QIAGEN, https://www.qiagen.com) to extract RNA from 200 μL clarified fecal suspensions pretreated with chloroform. After RNA extraction, we screened all samples for EV-D68 by real time reverse transcription PCR as described elsewhere (*2*). For molecular characterization, the viral protein 1 region was amplified by a nested PCR and sequenced as described elsewhere (*2*). The alignment and phylogenic analyses of sequences obtained after cleaning were performed using MEGA 7.0 software (https://www.megasoftware.net). 

We tested for EV-D68 in 567 fecal samples collected from 7 countries in West Africa: Cape Verde (n = 1), Gambia (n = 6), Guinea-Bissau (n = 5), Guinea Conakry (n = 391), Mauritania (n = 20), Niger (n = 85), and Senegal (n = 59), during June–September 2016. EV-D68 was detected in 16 (2.8%) patients from 3 countries: Guinea (11/391), Niger (1/85), and Senegal (4/59). The detection of EV-D68 in fecal samples from AFP patients in West Africa countries is consistent with case reports and case series from the United States, South America, Asia, and Europe during the same period (*7*–*11*). 

**Figure 1:**
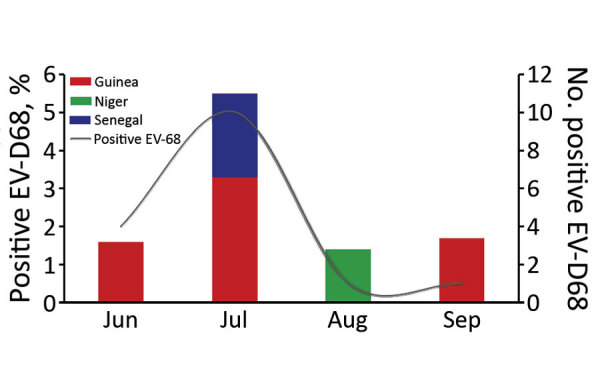
EV-D68 detection in fecal samples from patients with acute flaccid paralysis in 3 West Africa countries, June to September 2016. EV-D68, enterovirus D68.

**Figure 2:**
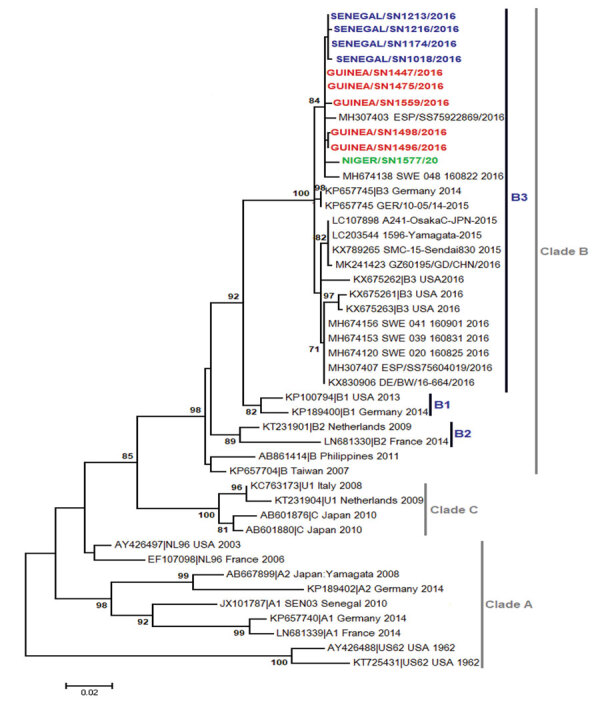
Phylogenetic relationships among EV-D68 strains detected in Guinea (red), Niger (green), and Senegal (blue), June–September 2016. We used the maximum-likelihood method based on the Tamura-Nei model method in MEGA7 (http://www.megasoftware.net) to generate the phylogenetic tree constructed on the viral protein 1 region of EV-D68 strains. Sequences are identified by GenBank accession number, country, and period of detection. The phylogenetic tree is rooted by the oldest EV-D68 sequence in GenBank, the Fermon strain. We performed 1,000 bootstrap replications to determine the consensus tree; support for nodes present in >70% of the trees are annotated. EV-D68, enterovirus D68.

The first EV-D68–associated AFP case was detected in Guinea in June 2016. Most cases (10/16) in West Africa in 2016 were detected during July (Figure 1), similar to the seasonality that has been observed in several other countries, including the United States (*12*), the Netherlands (*13*), and Senegal (*3*). Most EV-D68–positive patients (64.3%) were children <5 years old, consistent with our previous report from Senegal (*2*,*3*). BLAST analysis (https://blast.ncbi.nlm.nih.gov/Blast.cgi) showed that all sequenced EV-D68 strains shared >98% homology with strains detected in Spain, Sweden, Germany, Japan, and China. Phylogenetic analysis of the viral protein 1 fragment revealed that all sequences from West Africa belonged to clade B, subclade B3 (Figure 2). Indeed, EV-D68 subclade B3 was the predominant strain reported in several global regions during the same period (*9*–*14*). Moreover, results from phylogenetic testing showed that EV-D68 strains in West Africa clustered with strains circulating in Spain (GenBank accession no. MH307403) and Sweden (accession no. MH674138), with a bootstrap value of 97. 

Our study has some limitations. One ongoing issue is the inability to accurately describe the flaccid paralysis syndrome. Radiography imaging will probably help distinguish AFM from other AFP conditions. In addition, detecting EV-D68 in feces does not prove a causal relationship with AFM, although in this study all of the fecal samples tested negative for poliovirus and other enteroviruses, ruling out those possible alternative diagnoses. The absence of EV-D68 positive patients from the other West Africa countries may be due to the small number of samples collected and screened during the study period. EV-D68 prevalence in West Africa might be higher if respiratory samples, known to yield higher EV-D68 counts than fecal samples, were used for screening (*6*,*14*). Recently, the Pan American Health Organization and the World Health Organization provided updated recommendations to include respiratory sampling in suspected AFP cases (*15*). 

## Conclusions

This study provides evidence of more widespread EV-D68 circulation in West Africa in 2016 than previously reported. Enhanced surveillance for EV-D68, including collecting respiratory specimens from patients with confirmed cases of AFM, is needed to improve our understanding of this disease and its burden. Phylogeographic and phylodynamic studies based on full genomes are needed to better understand the introduction of EV-D68 in Africa during these different outbreaks. 
